# Multiple Target Tracking Based on Multiple Hypotheses Tracking and Modified Ensemble Kalman Filter in Multi-Sensor Fusion

**DOI:** 10.3390/s19143118

**Published:** 2019-07-15

**Authors:** Zequn Zhang, Kun Fu, Xian Sun, Wenjuan Ren

**Affiliations:** 1 Institute of Electronics, Chinese Academy of Sciences, Beijing 100190, China; 2 Key Laboratory of Technology in Geo-spatial Information Processing and Application System, Chinese Academy of Sciences, Beijing 100190, China

**Keywords:** Multi-sensor fusion, multiple target tracking, ensemble Kalman filter, multiple hypotheses tracking

## Abstract

In multi-sensor fusion (MSF), the integration of multi-sensor observation data with different observation errors to achieve more accurate positioning of the target has always been a research focus. In this study, a modified ensemble Kalman filter (EnKF) is presented to substitute the traditional Kalman filter (KF) in the multiple hypotheses tracking (MHT) to deal with the high nonlinearity that always shows up in multiple target tracking (MTT) problems. In addition, the multi-source observation data fusion is also realized by using the modified EnKF, which enables the low-precision observation data to be corrected by high-precision observation data, and the accuracy of the corrected data can be calibrated by the statistical information provided by the EnKF. Numerical studies are given to demonstrate the effectiveness of our proposed method and the results show that the MHT-EnKF method can achieve remarkable enhancement in dealing with nonlinear movement variation and positioning accuracy for MTT problems in MSF scenario.

## 1. Introduction

In multi-sensor surveillance systems like radar-based tracking, sonar-based tracking, and video-based tracking, multiple target tracking (MTT) is a vital problem that always arises. At the same time, the problems brought by multi-sensor monitoring (different positioning measurement methods lead to differences in the accuracy of various positioning information) have also arisen. Therefore, it is necessary to present a method to make use of the high-precision information in multi-source observation data to correct the low-accuracy information, that is, multi-sensor fusion (MSF).

Unlike some applications of the evidence theories for MSF in target recognition [1,2], the purpose of MTT in the MSF scenario is to simultaneously maintain the confirmed tracks for multiple targets and get more accurate estimates of the target trajectories. Once the tracks are generated and confirmed, the number of targets can be estimated and the parameters (velocity, acceleration, future predicted position, etc.) can be computed for each track. Therefore, there are two essential problems in MTT: (1) data/track association; and (2) state estimation/fusion.

The basic step of MTT is the measurement of track association. When a new group of measurements is generated by a sensor, the processing of each observation point can be divided into three situations: (1) assigned to an existing track, (2) considered as a new track, or (3) considered as a false alarm. The global nearest neighbor algorithm (GNN) [3] is one of the simplest and most widely spread approaches for target tracking association. The GNN simply considers the distances between measurements and tracks and associates those that fall closest to each other. More approaches are based on probabilistic methods (probabilistic data association (PDA) [4], joint probabilistic data association (JPDA) [5], nearest neighbor joint probabilistic data association (NNJPDA) [6], etc.). Take the JPDA algorithm as an example—firstly, multiple track hypotheses are generated in the JPDA, and then the probabilities of the hypotheses are calculated, and the assignment hypotheses of each track are merged. This means that the track states are updated by all the measurements within a track gate. Overall, these methods cannot appropriately manage the appearance or disappearance of tracks and make a so-called “hard” decision on the current observation scan in cases of conflicting measurements to track association hypotheses.

On the other hand, the problems that result from finding the best (or most likely) assignment of input measurements to existing tracks (as in the GNN) or choosing the best hypothesis (as in the JPDA method) have led to a prevalence of the multiple hypothesis tracking (MHT) approach. The MHT method forms alternative association hypotheses in case of observation of track conflict situations and utilizes sequential scans to solve the association problem, which anticipates that future observations will resolve the tracking assignment ambiguities. Generally, the MHT method includes two categories: (1) the hypothesis oriented MHT (HOMHT), and (2) the track oriented MHT (TOMHT). Singer et. al. [7] first presented the idea of propagating multiple assignment hypotheses. However, the HOMHT proposed by Reid [8] forms and expands a large number of hypotheses from scan to scan for data association. In contrary to the HOMHT, the TOMHT maintains track trees for incompatible tracks on last scans and prunes all the unreliable tree branches (track hypotheses) to obtain the best global hypothesis [9]. Recent research on MHT algorithms has mainly focused on improving the association algorithm in MHT [10,11,12,13,14]. However, the processing for the covariance setting and only solving linearity problems for the Kalman filter (KF) applied in the MHT state estimation/fusion step, especially when an MSF scenario shows up, is still left to be solved.

On the other hand, the main target state estimation/fusion methods include the KF, the extended Kalman filter (EKF), and the particle filter (PF), etc. The KF can achieve higher computational efficiency only when there are few system parameters. As mentioned before, the KF method is a kind of algorithm which makes use of the linear system state equation to estimate the state of the system by the input and output data of the system, so it cannot deal with non-linear problems [15]. The EKF method is a nonlinear version of the KF, but it is limited to dealing with weakly nonlinear problems with defined Jacobi matrices [16]. Septier et al. [17] uses Markov chain Monte Carlo (MCMC) techniques for multi-target tracking. The main problem with MCMC strategies is that they are iterative in nature and take an unknown number of iterations to converge. The PF algorithm is also based on the idea of Monte Carlo theory, in which the probability is expressed by the form of a particle set, but it requires a large number of samples to complete the calculation. Vehtari et al. [18] proposed a Rao-Blackwellized PF based EKF algorithm for MTT. However, this research still is still based on the assumption that the target dynamics are linear and the ability to solve nonlinear problems is limited by the EKF.

The ensemble Kalman Filtering method (EnKF) is another effective extension of the KF method used to deal with nonlinear problems [19]. Cui and Zhang [20] applied the EnKF method to a multi-sensor target tracking problem, in which the observation error of different sensors was assumed to have the same value. This study proved that the EnKF method is superior to the EKF method for the target tracking result. However, as the EnKF is not perfectly designed in this work, the nonlinearity caused by the coexistence of velocity and acceleration is not considered. Pornsarayouth et al. [21] used the EnKF method to deal with the problem of “out of sequence” measurements and verify that the EnKF method is superior to KF, PF and EKF in dealing with this kind of problem. However, this research assumes that the target motion keeps uniform velocity and does not realize the prediction of the subsequent trajectory. Zhang et al. [22] established an improved EnKF model to solve an asynchronous data fusion of a target tracking nonlinearity problem with the coexistence of velocity and acceleration. However, these studies have not extended the EnKF approach to MTT scenarios. Generally speaking, the application of the EnKF method in target trajectory analysis is comparatively preliminary.

In this study, we proposed a modified EnKF to substitute the KF in MHT to deal with the nonlinearity in the MTT and MFS scenarios. This allows us to consider the coupling of acceleration and velocity with nonlinear kinematic equations in each time interval. In addition, the multi-source observation data fusion is realized by using the EnKF, which enables the low-precision observation data to be corrected to high-precision observation data, and the accuracy of the corrected data can be directly calibrated by the statistical information provided by the EnKF results. In the remainder of this paper, we will first introduce the MHT in general terms (Section 2). Then, we will present the modification on the EnKF for the MHT and their combination for the MTT in the MSF scenario (Section 3). The experimental results and discussion are in Section 4. Finally, Section 5 concludes the paper.

## 2. Traditional MHT with Kalman Filter

In this study, the TOMHT is chosen for track hypotheses generation. In this section, we will present the basic concepts regarding TOMHT.

### Basic Concepts

In the TOMHT, the observation set at scan *k* is defined as:

(1) $$O_{k}=\left\{o_{k}^{0},\;o_{k}^{1},\;o_{k}^{2},\;o_{k}^{3},\;\ldots \;,\;o_{k}^{N_{k}}\right\}$$

where $N_{k}$ is the number of observations, $o_{k}^{i}$ (i≠0) is the *i*-th measurement in scan *k* and $o_{k}^{0}$ represents the missing detection or false alarm.

The track hypothesis matrix is defined as an ensemble of measurements:

(2) $$Z_{k}^{i}\;=\left\{z_{1}^{i_{1}},\;z_{2}^{i_{2}},\;z_{3}^{i_{3}},\;z_{4}^{i_{4}},\;\ldots \;,\;z_{k}^{i_{k}}\right\}$$

where $z_{j}^{i_{j}}$ is the $i_{j}$-th ($i_{j}\in \left\{1,\;\ldots \;,\;N_{j}\right\}$) measurement that is assumed to be associated to the *i*-th track at scan *j* (j∈{1, … , k}). The TOMHT aims to utilize the state estimation from the most probable global hypothesis for the track maintenance and keeps a track tree in a certain number of sequential scans to find the global hypothesis. Here, we assume that any two tracks are not allowed to originate from one common beginning and any two tracks in one global hypothesis do not share any common observations.

A track tree in the TOMHT contains multiple track branches, also known as track hypotheses. Each track maintained from previous scans may trigger several new tracks. In addition, a new track tree will be built for each measurement to consider the probability that each measurement is a new target (more details of TOMHT can be found in [23,24]).

In the TOMHT, each hypothesis is associated with a track score defined as a log likelihood ratio (LLR). The LLR score of $Z_{k}^{i}$ can be calculated as:

(3) $$L(Z_{k}^{i})\;=\;L(Z_{k-1}^{i})+\;\Delta L(Z_{k}^{i})$$

and the term $\Delta L(Z_{k}^{i})$ is defined as:

(4) $$\Delta L(Z_{k}^{i})=\{\begin{array}{c} \ln\left(1-{\hat{P}}_{D}\right);\;\;\;\;\;\;\;\;no\;update\;on\;scan\;k\\ \Delta L_{u}\left(k\right);\;\;\;\;\;track\;update\;on\;scan\;k\;\;\;\;\; \end{array}$$

The loss in track score when there is no update observation on scan *k* is a function of the expected detection probability ${\hat{P}}_{D}$, where ${\hat{P}}_{D}$ and $\Delta L_{u}\left(k\right)$ can be calculated using the original work in [24]. In addition, if a signal intensity (such as the signal-to-noise ratio (SNR)) is measured, it may also be added to the track score function.

The track score of the new hypothesis can be initialized as:

(5) $$L(Z_{1}^{i})\;=\ln\left(\frac{\lambda_{N}}{\lambda_{F}}\right)$$

where $\lambda_{F}$ denotes the spatial density of clutters and $\lambda_{N}$ denotes the spatial density of new targets, both of which are assumed to be Poisson distributed. After the individual track scores are calculated, the score of one certain hypothesis is the sum of all track scores contained in that hypothesis. Then, given the hypothesis scores, the most probable global hypotheses can be found out.

As a track may show up in several hypotheses, so that its probability is the sum of probabilities of all hypotheses which contain it, the classical sequential probability ratio test (SPRT) based on the current score of a certain track versus the upper and lower thresholds ($T_{low}$, $T_{up}$) characterizes the status of the track. Generally, a track will be confirmed as long as its score exceeds the upper threshold $T_{up}$ and will be directly deleted once its score falls below the lower threshold $T_{low}$. The upper and lower thresholds are defined as:

(6) $$T_{low}\;=\ln\left(\frac{\beta }{1-\alpha }\right),\;T_{up}\;=\ln\left(\frac{1-\beta }{\alpha }\right)$$

where α and β are the false and true track confirmation probability, respectively. If the track score falls between the upper and lower thresholds, the corresponding track is still tentative and is required to be further certified.

After a measurement has been associated with a track in the current scan, the state update is performed using the Kalman Filter (KF):

(7) $${\hat{x}}_{k|k}={\hat{x}}_{k|k-1}+K_{k}\left(z_{k}-H_{k}{\hat{x}}_{k|k}\right)$$

(8) $$P_{k|k}=\left(I-K_{k}H_{k}\right)P_{k|k-1}{\left(I-K_{k}H_{k}\right)}^{T}+K_{k}R_{k}K_{k}^{T}$$

where *K* is the Kalman gain, $P_{k|k}$ is the post error covariance, $P_{k|k-1}$ is the predicted error covariance, ${\hat{x}}_{k|k}$ is the posteriori state estimate at time *k* given observations up to and including at time *k* and $R_{k}$ is the error covariance of the observation noise. Then, the predict phase of the KF uses the state estimate from the current timestep to produce an estimate of the state at the next time step:

(9) $${\hat{x}}_{k+1|k}=F_{k}{\hat{x}}_{k|k}+B_{k}u_{k}+w_{k}$$

(10) $$P_{k+1|k}=F_{k}P_{k|k}F_{k}^{T}+Q_{k}$$

where $F_{k}$ is the previous state transition model, $B_{k}$ is the input-control model applied to the control vector $u_{k}$ and $w_{k}$ is the system noise which is drawn from a zero mean multivariate normal distribution with covariance $Q_{k}$.

## 3. TOMHT with Modified EnKF

The EnKF method is mainly divided into two steps: a forecast step and an update step. In the forecast step, first, a set of sample parameters is generated to predict future changes of the mode based on a priori information of the sample parameters. Each of these sample parameters is in the form of a model state vector that contains dynamic and static parameters and observations. In the update step, the state vector in the parameter sample set is corrected by comparing the difference between the predicted value obtained by the prediction step and the actual observation data. This section will modify the EnKF method to handle multi-source data fusion problems for the TOMHT.

### 3.1. Ensemble Matrix

An ensemble matrix *Y* is first introduced in the EnKF, of which each column represents each element in the sample set. Therefore, each column is defined as a state vector $y^{j}$, with *j* being the label of the state vector and the *j*-th column of the ensemble matrix. The state vector consists of dynamic and static parameters, which can be expressed as:

(11) $$Y_{k}=Y\left(t_{k}\right)=\left(\begin{array}{c} \eta^{1}\;\;\;\;\;\;\;\ldots \;\;\;\;\;\;\eta^{Ne}\\ \gamma^{1}\;\;\;\;\;\;\;\ldots \;\;\;\;\;\;\gamma^{Ne} \end{array}\right)$$

where *Ne* is the number of samples in the ensemble, $\eta^{i}$ represents the dynamic parameters, $\gamma^{i}$ represents static parameters and $t_{k}$ is the time of scan *k*. $\langle Y\left(t_{i}\right)\rangle$ is the mean of the ensemble matrix:

(12) $$\langle Y\left(t_{k}\right)\rangle =\;Y\left(t_{k}\right)1_{Ne}$$

where $1_{Ne}$ is a $N_{e}\times N_{e}$ matrix with each element being $1/N_{e}$. Then, the disturbance in the ensemble matrix *Y* can be expressed as:

(13) $$Y^{\prime }\left(t_{k}\right)=Y\left(t_{k}\right)-\langle Y(t_{k})\rangle$$

Then, the covariance matrix $C_{Y}$ for the sample ensemble is:

(14) $$C_{Y}\left(t_{k}\right)=\frac{Y^{\prime }\left(t_{k}\right)Y^{\prime }{\left(t_{k}\right)}^{T}}{Ne-1}$$

### 3.2. Observation Matrix

Meanwhile, the measurement vector $d\in R^{N_{d}}$ is introduced, where $N_{d}$ is the total number of observation values. We add a perturbation {$\varepsilon^{j}$} with zero mean to d to obtain the measurement matrix *D*:

(15) $$D\left(t_{k}\right)=\left(d^{1}\left(t_{k}\right),d^{2}\left(t_{k}\right),d^{3}\left(t_{k}\right),\ldots \;,d^{Ne}\left(t_{k}\right)\right)\in \;R^{N_{d}\times Ne}$$

where $\;d^{j}$ is defined as:

(16) $$\;d^{j}\left(t_{k}\right)=d\left(t_{k}\right)+\varepsilon^{j},\;\left(j=1\ldots Ne\right)$$

$d\left(t_{k}\right)$ is the observation vector at time $t_{k}$ of scan *k*. In the multi-sensor data fusion scenario, the perturbation or observation error {$\varepsilon^{j}$} will be different for each sensor. Thus, Equation (16) in the EnKF should be modified to meet the demands:

(17) $$\;d^{j}\left(t_{k}\right)=d\left(t_{k}\right)+\varepsilon_{n}^{j}\left(t_{k}\right),\;\;\left(j=1\ldots Ne\right)$$

where $\varepsilon_{n}^{j}\left(t_{k}\right)$ represents the perturbation for sensor *n* at time $t_{k}$. The perturbation matrix $E_{n}\left(t_{i}\right)$ of sensor *n* can be expressed as:

(18) $$E_{n}\left(t_{k}\right)=\left(\varepsilon_{n}^{1}\left(t_{k}\right),\varepsilon_{n}^{2}\left(t_{k}\right),\varepsilon_{n}^{3}\left(t_{k}\right),\ldots \;,\;\varepsilon_{n}^{Ne}\left(t_{k}\right)\right)\in \;R^{N_{d}\times Ne}$$

The observation covariance matrix of sensor *n* at time $t_{k}$ is:

(19) $$C_{D,n}\left(t_{k}\right)=\frac{E_{n}\left(t_{k}\right)E_{n}^{T}\left(t_{k}\right)}{Ne-1}$$

### 3.3. Update Step of the EnKF

The update for the ensemble $y_{j}^{a}$ is represented as follows:

(20) $$y_{j}^{a}=y_{j}^{\prime }+C_{Y}H^{T}{\left(HC_{Y}H^{T}+C_{D}\right)}^{-1}D_{j}^{\prime }$$

where$\;C_{Y}$ and $C_{D}$ are the covariance matrices of *Y* and *D*, $y_{j}^{a}$ is the updated state vector, and $y_{j}^{\prime }$ is the predicted state vector obtained from previous timestep. $D_{j}^{\prime }$ is defined as:

(21) $$D_{j}^{\prime }=d^{j}-Hy_{j}^{\prime }$$

In the multi-sensor data fusion scenario, Equation (20) and Equation (21) should be modified as:

(22) $$y_{j}^{a}\left(t_{k}\right)=y_{j}^{\prime }\left(t_{k}\right)+C_{Y}\left(t_{k}\right)H^{T}{\left(HC_{Y}\left(t_{k}\right)H^{T}+C_{D,k}\left(t_{k}\right)\right)}^{-1}D_{j}^{\prime }\left(t_{k}\right)$$

(23) $$D_{j}^{\prime }\left(t_{k}\right)=d^{j}\left(t_{k}\right)-Hy_{j}^{\prime }\left(t_{k}\right)$$

where the matrix *H* is a mapping matrix between the model parameter state vector $y_{j}^{\prime }$ and the observation vector $d^{j}$. Usually, there is no linear mapping between the model parameter state vector ${y_{j}}^{\prime }$ and the observation vector $d^{j}$ due to the strong nonlinearity of the model. This is the reason why the KF is unable to deal with nonlinear problems. However, when the predicted values of the observation data are contained in the elements of the state vector ${y_{j}}^{\prime }$, the form of the mapping matrix will be very simple. The elements in the mapping matrix H will only include 0 and 1:

(24) H=[O|I]

where *I* is a $N_{d}\times N_{d}$ identity matrix and *O* is a $N_{d}\times (N_{y}-N_{d})$ zero matrix.

### 3.4. Forecast Step of the EnKF

In this study, we no longer assume that the target motion is uniform during each time interval, so the state parameters are the target position (*xs*, *ys*), velocity (*xv*, *yv*), and acceleration (*xa*, *ya*). The state vector $y^{j}$ at time $t_{k-1}$ is expressed as:

(25) $$y^{j}(t_{k-1})=\left[\;xs^{j}(t_{k-1}),\;ys^{j}(t_{k-1}),\;xv^{j}(t_{k-1}),\;yv^{j}(t_{k-1})\;,xa^{j}(t_{k-1}),ya^{j}(t_{k-1})\right]$$

The predicted state vector $y^{j^{\prime }}$ at time $t_{k}$ is expressed as:

(26) $$y^{j^{\prime }}(t_{k})=\left[\;xs^{j^{\prime }}(t_{k}),\;xy^{j^{\prime }}(t_{k}),\;xv^{j^{\prime }}(t_{k}),\;yv^{j^{\prime }}(t_{k}),xa^{j^{\prime }}(t_{k}),ya^{j^{\prime }}(t_{k})\right]$$

Let Δt represent the time interval between time $t_{k}$ and $t_{k-1}$. The nonlinear dynamic governing equation used in this study is expressed as:

(27) $$\begin{array}{c} xs^{j^{\prime }}(t_{k})=xs^{j}(t_{k-1})+xv^{j}(t_{k-1})\cdot \Delta t+0.5\cdot xa^{j}(t_{k-1})\cdot \Delta t^{2}\\ ys^{j^{\prime }}(t_{k})=ys^{j}(t_{k-1})+yv^{j}(t_{k-1})\cdot \Delta t+0.5\cdot ya^{j}(t_{k-1})\cdot \Delta t^{2}\\ xv^{j^{\prime }}(t_{k})=xv^{j}(t_{k-1})+xa^{j}(t_{k-1})\cdot \Delta t\\ yv^{j^{\prime }}(t_{k})=yv^{j}(t_{k-1})+ya^{j}(t_{k-1})\cdot \Delta t\\ xa^{j^{\prime }}(t_{k})=xa^{j}(t_{k-1})\\ ya^{j^{\prime }}(t_{k})=ya^{j}(t_{k-1}) \end{array}\}$$

Although the predicted value of the acceleration value in Equation (27) is tentatively set to the acceleration value at the previous time, this acceleration is continuously corrected in the EnKF update step. After obtaining the predicted state vector $y^{j^{\prime }}$, Equation (20) can be used to update the $y^{j^{\prime }}$.

As the observation data of each time step continuously enters the EnKF system (history fitting process), the parameters (speed and acceleration) in the state vector $y^{j}$ can be gradually corrected and gradually approaches the true value.

Finally, by substituting Equation (20) to Equation (27) for Equation (7) to Equation (10) in TOMHT, the model system of the TOMHT based on the EnKF (MHT-EnKF) is established and can be applied to solve nonlinear movements in multi-sensor data fusion scenarios.

## 4. Experiments

### 4.1. Experimental Settings

To evaluate the performance of the proposed MHT-EnKF model, we design the following simulation scenarios. The first case is based on multi-source observation data of one moving target with acceleration. During the actual movement, the target enjoys a uniform acceleration motion with $v_{x,0}=5\;\mathrm{km}/h,\;a_{x}=0.2\;\mathrm{km}/h^{2},\;v_{y,0}=4\;\mathrm{km}/h,\;a_{y}=-0.2\;\mathrm{km}/h^{2}$. The observation data has four different sources with corresponding standard deviations of 2 km, 5 km, 8 km, and 15 km. Through the first case, we tend to verify the data fusion effectiveness of the MHT-EnKF algorithm in dealing with the MSF problem. The second case is presented with a set of multi-source observation data of three moving targets. Each target is moving at a different uniform speed ($\mathrm{Track}\;1:\;v_{x}=4\;\mathrm{km}/h,\;v_{y}=4\;\mathrm{km}/h$; $\mathrm{Track}\;2:\;v_{x}=5\;\mathrm{km}/h,\;v_{y}=3\;\mathrm{km}/h$; $\mathrm{Track}\;3:\;v_{x}=6\;\mathrm{km}/h,\;v_{y}=2\;\mathrm{km}/h)$. The observation data has six different sources with corresponding standard deviations of 0.5 km, 1 km, 1.5 km, 2 km, 3 km, and 5 km. To verify the capability of the MHT-EnKF of seeking out correct tracks through multiple track hypotheses, a set of randomly distributed false alarms are simultaneously generated with true observation data.

### 4.2. Performance Evaluation of Data Fusion Effectiveness of the MHT-EnKF

**Figure 1:**
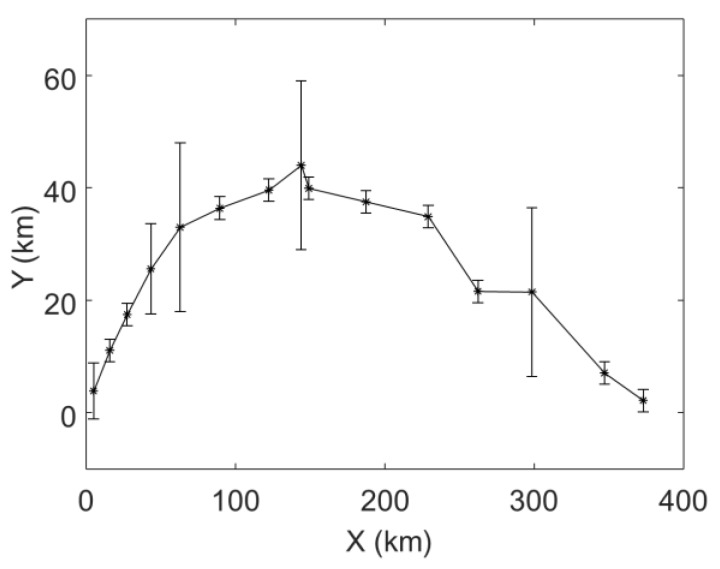
The trajectory and the error bar of each observation data.

**Figure 2:**
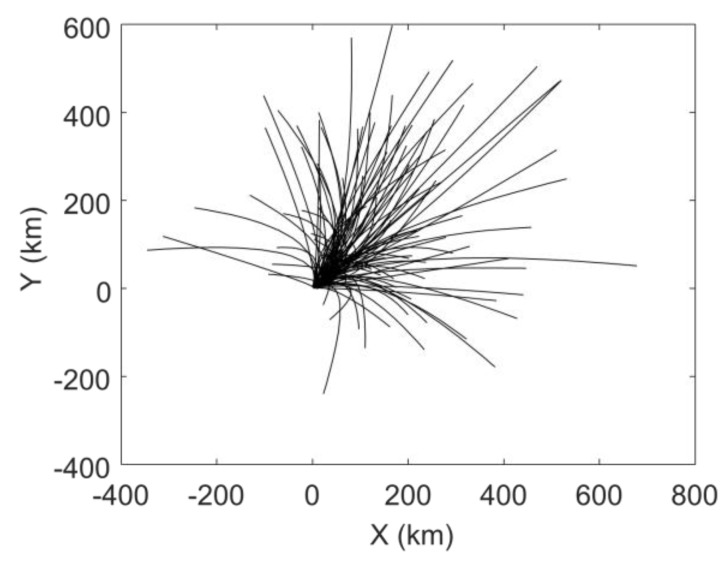
The target motion prediction based on initial velocity and acceleration guesses.

Figure 1 depicts the trajectory and the error bar of each observation data; the target trajectory has 15 data points. The larger the length of the error bar, the larger the standard deviation of the observation, which corresponds to 1 km, 2 km, 4 km and 8 km. The mean value of the observation error at each point is set to be zero. In the MHT-EnKF, the initial guess of the velocity and acceleration are set as follows: the initial velocity average in the X direction is set to be 6 km/h, and the standard deviation is 5 km/h; the initial average velocity in the Y direction is set to be 3 km/h, and the standard deviation is also 5 km/h; the initial average acceleration in the X direction and in the Y direction are all set to be 0 km/h^2^ with standard deviation of 0.25 km/h^2^. The number of EnKF ensembles is set to 100. Figure 2 is a prediction of the target motion trajectory based on initial velocity and acceleration guesses. It can be seen that the target predicted trajectory distribution without EnKF is discrete, and the actual motion state cannot be characterized.

**Figure 3:**
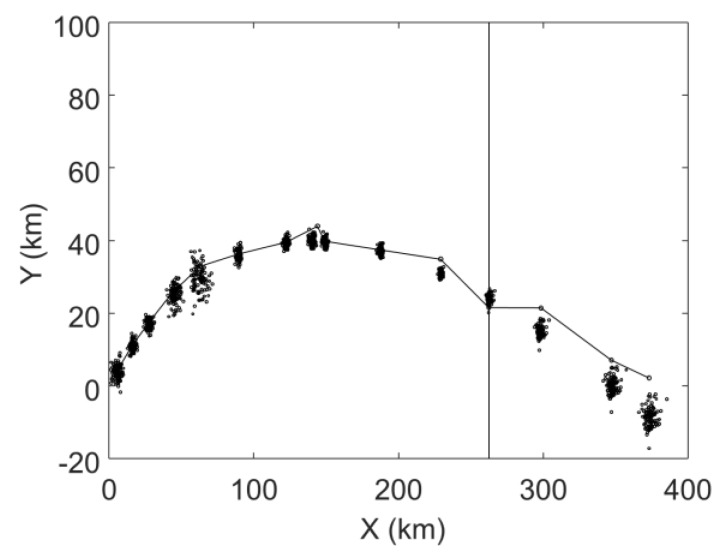
The matching results of the multiple hypotheses tracking-ensemble Kalman filter (MHT-EnKF).

**Figure 4:**
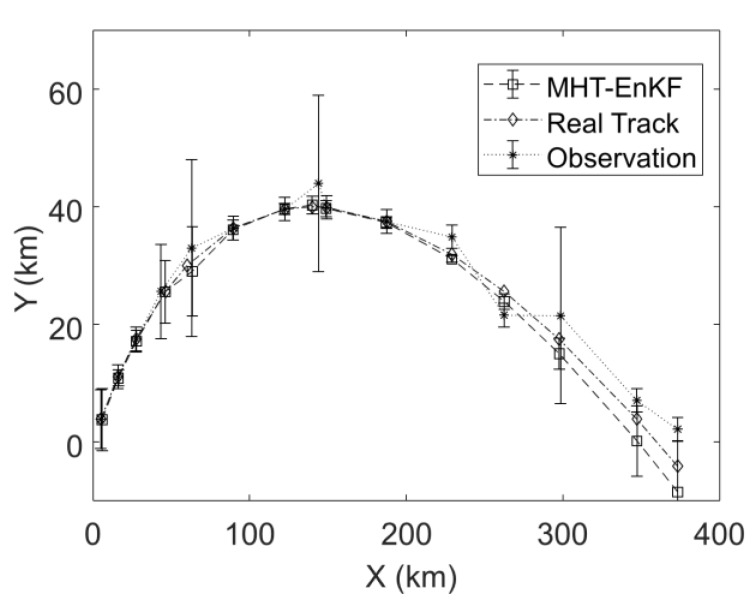
Comparison of the statistical information of 100 samples of the MHT-EnKF fitting result with the actual multi-source observation data.

Figure 3 shows the matching results of the MHT-EnKF based on the first 12 target observation data. The black curve represents the trajectory formed by the actual observation data. The black circle around each actual observation data is the 100 samples of the EnKF update values. It can be seen that the EnKF trajectory points represented by the black dots in Figure 3 have converged around each observation point, but there is still some deviation at several observation positions. We will use Figure 4 to explain this phenomenon.

Figure 4 is a comparison of the statistical information of 100 samples of the MHT-EnKF fitting results with the actual multi-source observation data. Since the EnKF utilizes the Monte Carlo idea to generate random samples, the corresponding statistical information (mean, variance, etc.) of the samples can be directly calculated. The dashed line marked by the square represents the EnKF fitting trajectory and the dashed line marked by “*” represents multi-source observation data. The midpoint of each line segment represents the mean of 100 samples of the EnKF or the observation, and the length of the line segment is proportional to the standard deviation. The dotted line with the diamond mark is the actual target motion trajectory. It can be seen from Figure 4 that after the EnKF update step, the results of the EnKF are not only closer to the actual target motion trajectory, but the standard deviation results of the EnKF trajectory also show a significant decrease, especially at several observation points with large observation errors (starting from the left, point 4, 5, 8, 11, 12), which means the EnKF results have a higher positioning accuracy. This is because the updated value of each time has two sources: one source is the current time prediction value obtained by the EnKF prediction step based on the sample update value of the previous time step, and the other source is the observation data at the current moment. When the accuracy of the observation data at the two moments is different, the calculation process of the EnKF makes it possible to correct the low-precision observation data with high-precision observation data and realize information fusion between different observation data sources. At the same time, the last three data points in Figure 3 and Figure 4 are the predicted results of the trajectory based on the target speed and acceleration matching results. It can be seen that the MHT-EnKF prediction value can still keep close to the real track.

**Figure 5:**
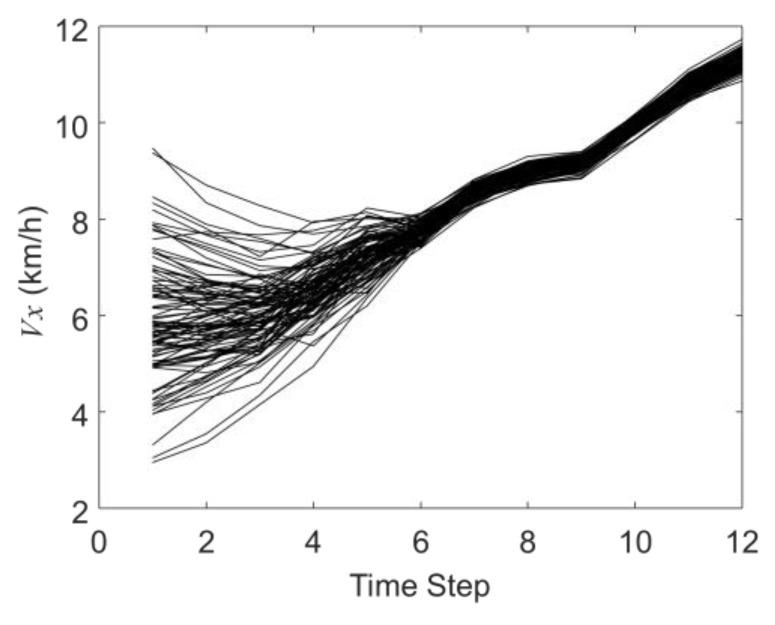
The history matching results of the velocity in the X-direction (*V_x_*).

**Figure 6:**
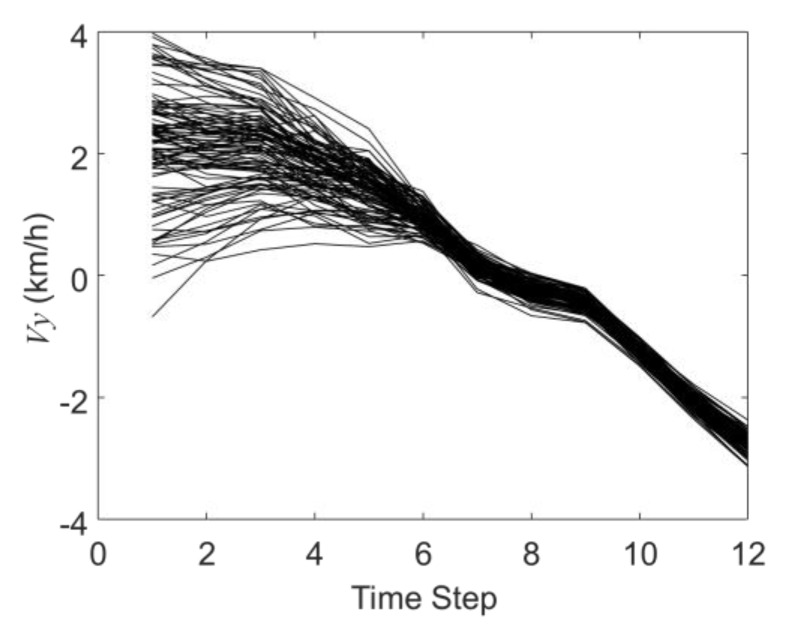
The history matching results of the velocity in the Y-direction (*V_y_*).

Figure 5 and Figure 6 show the history matching results of the velocities in the X-direction and Y-direction through the EnKF, in which the black curves represent 100 samples in the EnKF. It shows that the 100 curves gradually converge from the discrete distribution in the initial step to the last step. In the twelfth time step, the average speed in the X direction is 11.56 km/h, and the average speed in the Y direction is −2.93 km/h (the actual X direction speed is 11.80 km/h and the Y direction speed is −2.80 km/h).

**Figure 7:**
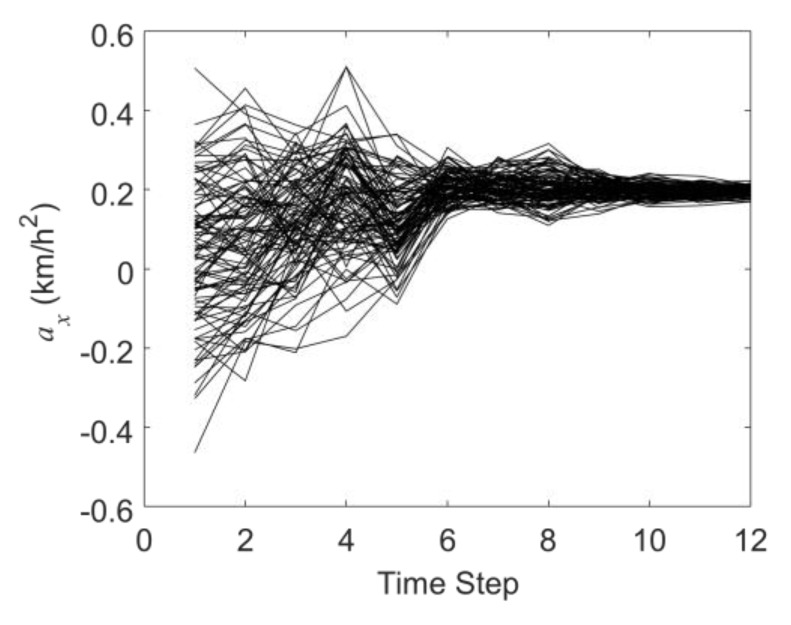
The history matching results of the acceleration in the X-direction (*a_x_*).

**Figure 8:**
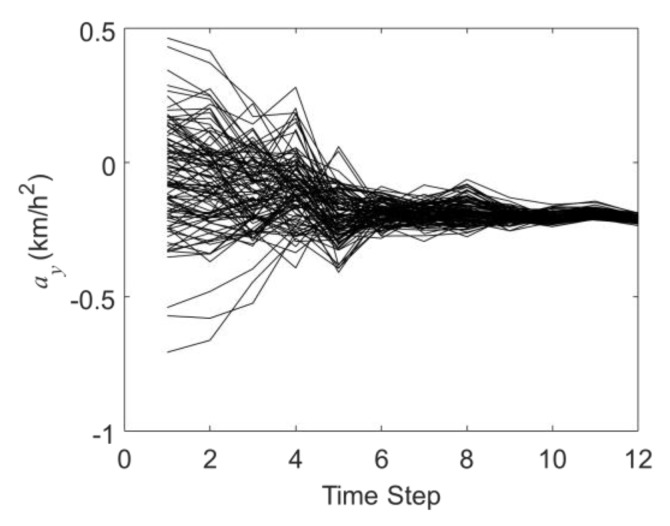
The history matching results of the acceleration in the Y-direction (*a_y_*).

Figure 7 and Figure 8 are the history matching results of the accelerations in the X-direction and Y-direction through the EnKF. It can be seen that as the EnKF proceeds, the black curves in Figure 7 and Figure 8 also gradually converge from the state of being largely discrete in the initial guess. At the twelfth time step, the average acceleration values in the X and Y directions obtained by the EnKF are 0.196 km/h^2^ and −0.211km/h^2^, respectively (the actual acceleration values are 0.2 km/h^2^ and −0.2 km/h^2^). This proves that in the MSF scenario, the MHT-EnKF model constructed in this study can match the accurate motion parameter values.

**Figure 9:**
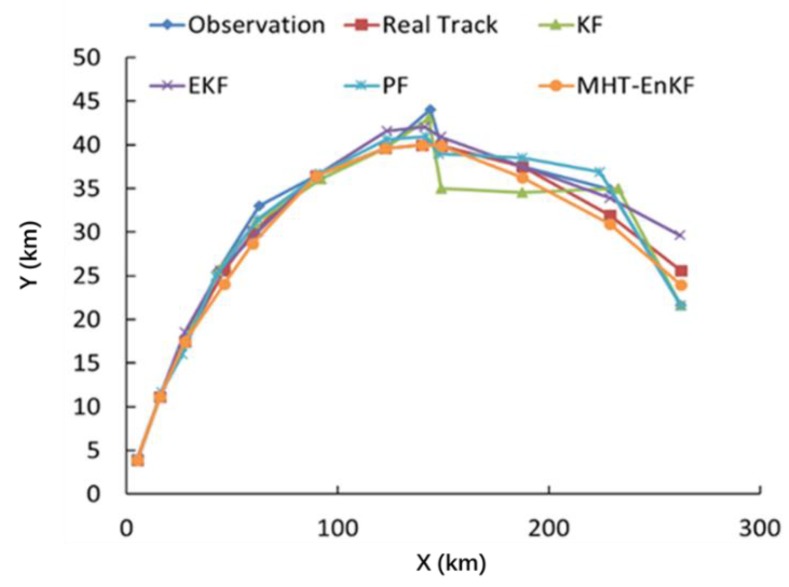
A comparison of the results of the trajectory fitting results based on the improved EnKF method with KF, EKF and PF.

**Figure 10:**
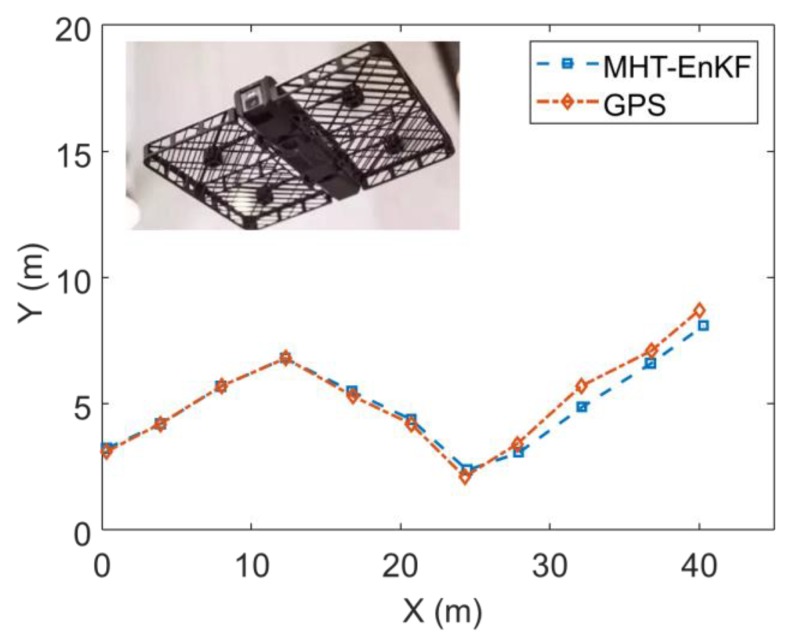
The MHT-EnKF tracking results for the tracking of a drone.

Figure 9 is a comparison of the trajectory fitting results based on the improved EnKF method with KF, EKF and PF. It can be seen from Figure 9 and Table 1 that the trajectory fitting result based on the MHT-EnKF method constructed in this study is closest to the actual trajectory and the other three methods show weak robustness when facing the perturbation in the observation series. It is also verified that the MHT-EnKF model constructed in this study has a robustness to deal with nonlinear motion problems. To further verify the robustness of the algorithm, we applied the algorithm to a piece of trajectory data we collected from a drone produced by Hover. As the GPS update frequency is 10 Hz with a positioning accuracy of 0.5 m, we selected 1 s as the time interval to obtain the positioning information. The tracking results are depicted in Figure 10. It can be seen from the figure that for the real target tracking problem, our algorithm fitting results can still keep close to observation trajectory.

### 4.3. Performance Evaluation of Multi-Target Tracking Effectiveness of the MHT-EnKF

In Section 4.2, we verified the superiority of the newly proposed MHT-EnKF model in multi-sensor data fusion. However, there is only one moving target in the whole monitoring and tracking process, which means that the advantages of the MHT are not really used. In this section, we design a multi-target and multi-sensor tracking scenario, in which the observation data in different scans comes from different sensors. This time, the initial guess of the velocity and acceleration in the MHT-EnKF are set as follows: the initial velocity average in the X direction is set to be 2 km/h with a standard deviation of 2.5 km/h; the initial average velocity in the Y direction is set to be 6 km/h with a standard deviation of 2.5 km/h; and the initial average accelerations in the X direction and in the Y direction are all set to be 0.5 km/h^2^ and −0.5 km/h^2^ with a standard deviation of 0.5 km/h^2^. The number EnKF ensembles is also set to 100. As the actual movement of each target keeps a uniform velocity, we will test the effectiveness of MHT-EnKF by whether the model will find the final acceleration to be 0 km/h^2^ or not.

**Figure 11:**
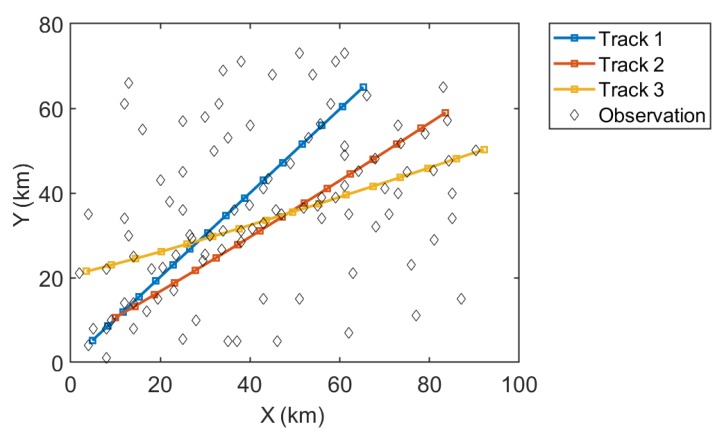
The tracking results of the MHT-EnKF for three targets.

**Figure 12:**
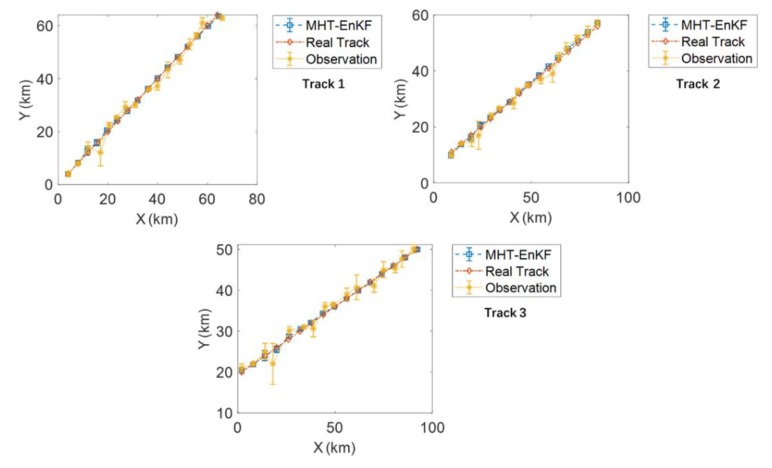
The comparison between the actual tracks, the corresponding observation points and the MHT-EnKF tracking results of Track 1, Track 2 and Track 3.

Figure 11 shows the tracking results of the MHT-EnKF for three targets. The observation data are represented by the randomly distributed diamond points. The false alarms and target observations are all included in these observation points without any distinction. We can see that the MHT-EnKF method seeks out three tracks out of these points, which are plotted as the red, yellow and blue lines. In order to test the correctness of our tracking results, Figure 12 depicts the comparisons between the actual tracks, the corresponding observation points and the MHT-EnKF tracking results with the statistical errors for each track. It shows that, as in the previous example, accuracy corrections can be formed between observation sources of different precisions, and the fusion positioning resulting is closer to the real tracks.

**Figure 13:**
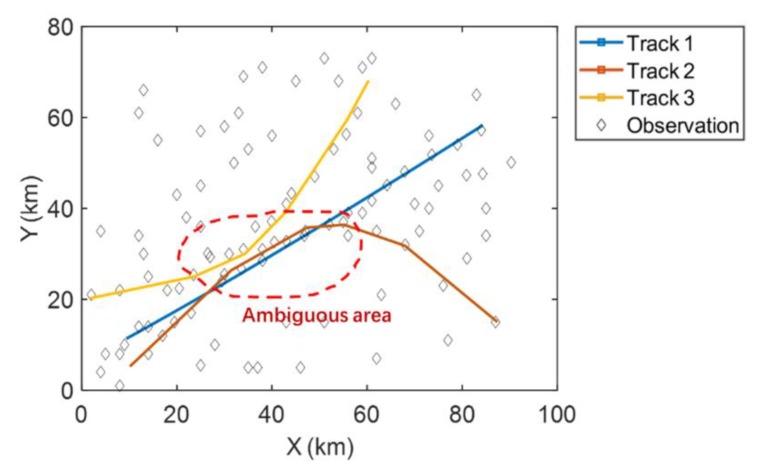
The tracking results of the EnKF for three targets without MHT.

In contrast, as false alarms are generated in each time step, an EnKF tracking process without applying MHT is depicted in Figure 13. We can see how the three tracks are getting closer until they reach the ambiguous area circled in red and tracking results gradually deviate from real ones shown in Figure 12 after they leave this area. In the end, even though the initial settings of the EnKF tracking without MHT are the same as those in Figure 11, the tracking results are totally different. This means that the EnKF tracking process without applying MHT is unable to detect real tracks when false alarms are disturbing the eyesight. The comparison between Figure 11 and Figure 13 shows the capability of MHT-EnKF in seeking real tracks when interference items appear.

**Figure 14:**
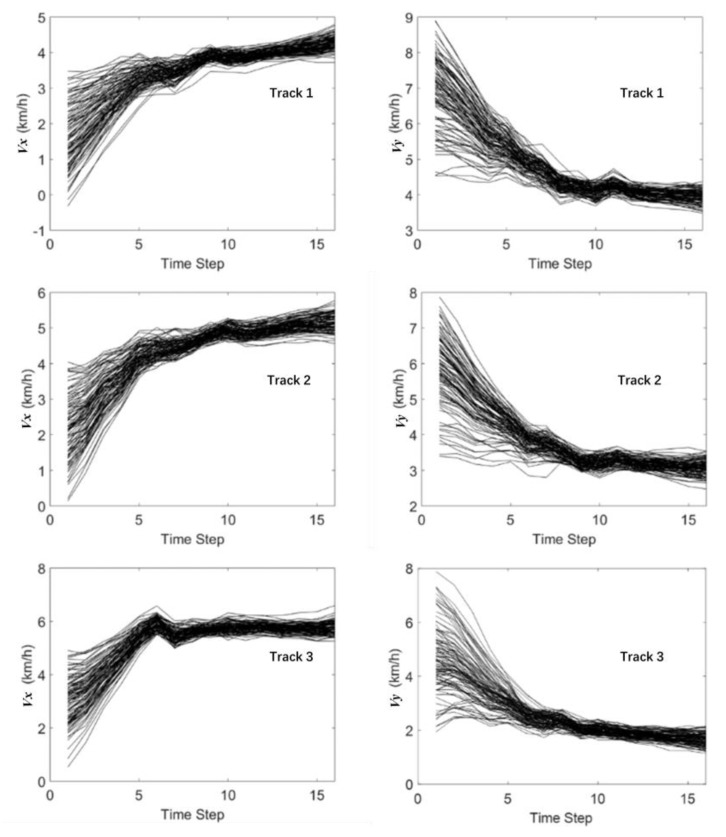
The history matching results of the velocity in the X-direction and Y-direction through the MHT-EnKF.

**Figure 15:**
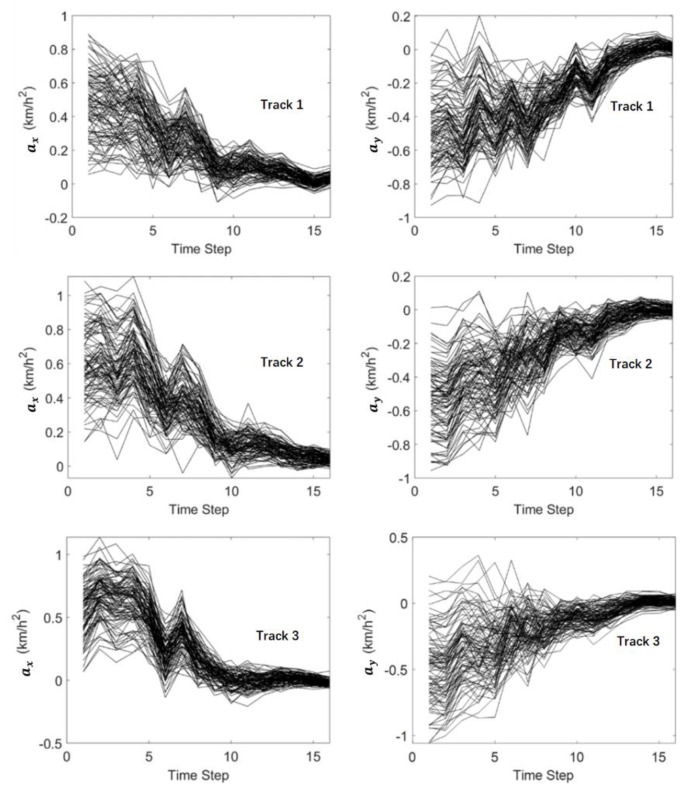
The history matching results of the acceleration in the X-direction and Y-direction through the MHT-EnKF.

Figure 14 and Figure 15 show the history matching results of the velocities and accelerations in the X-direction and Y-direction through the MHT-EnKF for three tracks. The black curves represent 100 samples in the EnKF. It shows that the 100 curves gradually converge from the discrete distribution in the initial step to the last step in Figure 14 and Figure 15. The final matching results are shown in Table 2. The history matching results are very close to the actual values. Moreover, even if the initial guess value deviates from the actual motion situation, the real trajectory can still be found in the case of interference with the false alarm condition. This proves the robustness of the MHT-EnKF method in dealing with MTT and MSF problems.

## 5. Conclusions

Target trajectory analysis based on multi-sensor detection data has always been one of the research focuses in the field of target tracking. In this paper, the EnKF method is modified to solve the MSF problem and a set of nonlinear kinematics equations in the forecast step of the EnKF are constructed, which makes the modified EnKF method capable of dealing with the nonlinearity of the coupling of velocity and acceleration. Furthermore, the MHT is introduced to help the EnKF to cope with the multi-target tracking problem. Thus, unlike former research, the MHT-EnKF method established in this study is able deal with nonlinear MTT problems in MSF scenarios.

Meanwhile, the feasibility of applying the MHT-EnKF model in MSF scenarios is verified by two simulation case studies. The following conclusions can be drawn: (1) in the MSF scenario, the motion state (velocity and acceleration) of the target can be accurately fitted by the MHT-EnKF based on the historical trajectory of the target and used to predict the future motion trajectory; (2) by adding different error disturbances to the observation data with different accuracies in the calculation process, the MHT-EnKF method can directly express the uncertainty of the multi-source observation data and take these uncertainty into account in the parameter fitting process; (3) the MHT-EnKF method can realize the fusion of multi-source observation data with different accuracies even for multi-target tracking problems and use high-precision observation data to improve the accuracy of low-precision observation data.

In the end, as the MHT-EnKF model can match the motion state parameters (velocity and acceleration) accurately, the abnormal changes of the target motion state parameters can be monitored. It should be mentioned that this work is not a real time computation. However, as the EnKF is a sequential method, once new data are available, these data can be used to update all parameters. This makes the EnKF suitable for real time computation. Some studies have been applied to solve the real time computation problems for the EnKF using distributed or parallel computing techniques. In addition, the data fusion considered in this study is a multi-sensor asynchronous data fusion problem, which means there is only one sensor monitoring each observation step. Thus, further research can be carried out for real time computing problems for the MHT-EnKF or the multi-sensor synchronous observation data fusion problem when one target may be simultaneously detected by several sensors.

## Figures and Tables

**Table 1:** The RMSE value of the Kalman filter (KF), extended Kalman filter (EKF), particle filter (PF) and EnKF (units: km).

Metrics	KF	EKF	PF	EnKF
**RMSE**	3.18	2.33	2.34	0.56

**Table 2:** History matching results of the accelerations and velocities in the X-direction and Y-direction (units: km/h).

Value	Track 1	Track 2	Track 3
$v_{x}$	4.08	4.92	5.87
$v_{y}$	3.91	3.11	1.90
$a_{x}$	0.07	0.03	−0.08
$a_{x}$	−0.06	0.05	0.01

## References

[1] Song Y., Wang X., Zhu J., Lei L. (2018). Sensor dynamic reliability evaluation based on evidence theory and intuitionistic fuzzy sets. Appl. Intell..

[2] Xiao F. (2019). Multi-sensor data fusion based on the belief divergence measure of evidences and the belief entropy. Inf. Fusion.

[3] Yi J., Du Y., Liang F., Zhou C. (2017). An auto-tracking algorithm for mesoscale eddies using global nearest neighbor filter. Limnol. Oceanogr. Methods.

[4] Rasmussen C., Hager G.D. (2001). Probabilistic data association methods for tracking complex visual objects. IEEE Trans. Pattern Anal. Mach. Intell..

[5] Fortmann T.E., Bar-Shalom Y., Scheffe M. (1983). Sonar Tracking of Multiple Targets Using Joint Probabilistic Data Association. IEEE J. Ocean. Eng..

[6] Varghese S., Sinchu P., Bhai D.S. (2016). Tracking Crossing Targets in Passive Sonars Using NNJPDA. Procedia Comput. Sci..

[7] Singer R.A., Sea R.G., Housewright K.B. (1974). Derivation and evaluation of improved tracking filter for use in dense multitarget environments. IEEE Trans. Inf. Theory.

[8] Reid D.B. (1979). An Algorithm for Tracking Multiple Targets. IEEE Trans. Automat..

[9] Kosuge Y., Tachibana Y., Tsujimichi S. (2015). A track-oriented multiple hypothesis multitarget tracking algorithm. Elec. Commun. Jpn..

[10] He S., Shin H.S., Tsourdos A. (2018). Track-Oriented Multiple Hypothesis Tracking Based on Tabu Search and Gibbs Sampling. IEEE Sens. J..

[11] Hyunhak S., Bonhwa K., Jill N. (2018). Robust Target Tracking with Multi-Static Sensors under Insufficient TDOA Information. Sensors.

[12] Yao L., Liu Y., He Y. (2018). A Novel Ship-Tracking Method for GF-4 Satellite Sequential Images. Sensors.

[13] Gruyer D., Demmel S., Magnier V. (2016). Multi-Hypotheses Tracking using the Dempster-Shafer Theory, application to ambiguous road context. Inf. Fusion.

[14] Thomaidis G., Tsogas M., Lytrivis P. (2013). Multiple hypothesis tracking for data association in vehicular networks. Inf. Fusion.

[15] Ge Q., Wei Z., Cheng T., Chen S., Wang X. (2017). Flexible Fusion Structure-Based Performance Optimization Learning for Multisensor Target Tracking. Sensors.

[16] Bolognani S., Oboe R., Zigliotto M. (1999). Sensorless full-digital pmsm drive with ekf estimation of speed and rotor position. IEEE Trans. Ind. Electron..

[17] Septier F., Carmi A., Pang S.K. (2009). Multiple Object Tracking Using Evolutionary MCMC-Based Particle Algorithms. IFAC Proc. Vol..

[18] Verlaan M., Heemink A. (2001). Nonlinearity in data assimilation applications: A practical method for analysis. Mon. Weather Rev..

[19] Evensen G. (2003). The Ensemble Kalman Filter: Theoretical formulation and practical implementation. Ocean Dyn..

[20] Cui B., Zhang J. (2008). The Improved Ensemble Kalman Filter for Multisensor Target Tracking. Int. Symp. Inf. Sci. Eng..

[21] Pornsarayouth S., Wongsaisuwan M., Yamakita M. (2011). An Improvement of Ensemble Kalman Filter for OOSM Tracking. IFAC Proc. Vol..

[22] Zhang Z., Ren W., Fu K., Fang J., Zhang Y. (2018). Research on Multi-source and Asynchronous Data Fusion of Target Trajectory Based on the Modified Ensemble Kalman Filter Method. J. Electon. Inf. Technol..

[23] Sun J., Li Q., Zhang X. (2017). An Efficient Implementation of Track-Oriented Multiple Hypothesis Tracker Using Graphical Model Approaches. Math. Probl. Eng..

[24] Blackman S. (2004). Multiple hypothesis tracking for multiple target tracking. IEEE Aerosp. Electron. Syst. Mag..

